# Efficacy and Safety of Oral Metronomic Chemotherapy in Recurrent Refractory Advanced Gynaecological Cancer: An Experience From the Regional Cancer Centre of Eastern India

**DOI:** 10.7759/cureus.53232

**Published:** 2024-01-30

**Authors:** Ranti Ghosh, Kalyan Kusum Mukherjee, Ranajit Mandal, Tapas Maji, Debarshi Lahiri, Suparna Mazumder, Bodhisattwa Dutta, Debjit Ghosh, Jayanta Chakrabarti

**Affiliations:** 1 Radiation Oncology, Chittaranjan National Cancer Institute, Kolkata, IND; 2 Medical Oncology, Chittaranjan National Cancer Institute, Kolkata, IND; 3 Gynecologic Oncology, Chittaranjan National Cancer Institute, Kolkata, IND; 4 Radiodiagnosis, Chittaranjan National Cancer Institute, Kolkata, IND; 5 Surgical Oncology, Chittaranjan National Cancer Institute, Kolkata, IND

**Keywords:** survival, safety, outcome, oral metronomic, gynaecological cancer

## Abstract

Introduction: The outcome of recurrent/metastatic gynaecological malignancy has drastically improved with the introduction of poly(ADP-ribose) polymerase inhibitors and immunotherapy, but the use of these drugs in routine practice is complicated due to access barriers and their high cost in developing countries. The purpose of this study is to present the clinical response, outcome and safety of oral metronomic chemotherapy (OMCT) in resource-limited, financially constrained populations.

Methods: This is a retrospective study on patients with advanced gynaecological cancer treated at Chittaranjan National Cancer Institute, Kolkata, India, from 2021 to 2023. The patients were treated with one of these two regimens: a split-dose course of cyclophosphamide (50 mg orally once daily for 21 days) and capecitabine (500 mg twice daily continuous) or a fixed-dose combination (capecitabine 1800 mg and cyclophosphamide 80 mg orally for 14 days in every 21 days) until disease progression or unacceptable toxicities occurred. All data was captured from the hospital's medical records until June 2023. Toxicity data was reported per the Common Terminology Criteria for Adverse Events (CTCAE) v5.1, and progression-free survival (PFS) was estimated using Kaplan-Meier methods.

Results: Among 34 screened patients, 10 were excluded due to noncompliance. This study analysed 24 patients with a median age at diagnosis of 56 years (IQ range 44-75). Sixteen (67%) patients were at stage IV disease with an Eastern Cooperative Oncology Group (ECOG) performance status of 3. Ovarian and cervical cancers were 80% and 20%, respectively; among them, 16 (67%) patients were platinum-refractory. Forty-two per cent of patients received three lines of chemotherapy before OMCT. A split course versus fixed dose was given to 67% versus 33% of the population; the best responses per the Response Evaluation Criteria in Solid Tumours v1.1 were complete response in 12%, partial response in 67% and stable disease in 21%. The most common toxicities were grade I anaemia (54%), grade I chemotherapy-induced nausea and vomiting (46%), grade I fatigue (42%) and grade I neutropenia (21%). Twenty-five per cent of patients were offered next-line systemic therapy after progression. The entire cohort had a median PFS of nine months (95%, CI: 5.2-12.7). Cox regression analysis identified a median PFS of 12 months (95%, CI: 6.2-17.7) among platinum-refractory groups.

Conclusion: OMCT was a well-tolerated, affordable regimen with durable clinical response and survival outcome (median PFS of nine months) in recurrent, refractory advanced gynaecological cancer and can be offered to patients at resource-limited centres.

## Introduction

Ovarian and cervical cancers are the most common gynaecological cancers affecting women worldwide. Although cervical cancer is on the decline, it remains the second most common cancer in women after breast cancer [1]. Ovarian cancer is the eighth most common malignancy in women and the eighth most common cause of death from cancer globally [2]. Advanced-stage ovarian cancer is associated with high morbidity and mortality. Despite an initial good response to chemotherapy, overall survival (OS) in advanced epithelial ovarian carcinoma is not encouraging. Five-year survival remains only 27% [3]. The leading cause of treatment failure is recurrence, which may be platinum-sensitive (platinum-free interval of more than six months) or platinum-resistant disease (platinum-free interval of less than six months). Progression-free survival (PFS) shortens with each subsequent relapse, particularly in platinum-resistant disease. The addition of bevacizumab, a poly(ADP-ribose) polymerase (PARP) inhibitor in recurrent settings, results in encouraging improvement in survival rates. Unfortunately, in cervical cancer, the recurrence rates are 10-20% in early-stage disease and up to 70% in locally advanced disease. Only 10-15% of recurrent patients live longer than 12 months, even if optimal treatment is provided [4]. Recently, bevacizumab immunotherapy has been approved in recurrent settings along with chemotherapy, dramatically improving OS. But most of the time, advanced, metastatic, recurrent epithelial ovarian and cervical cancer patients present with poor performance status and are financially exhausted after multiple lines of treatment. The accessibility and effectiveness of bevacizumab, PARP inhibitor and immunotherapy are limited in this setting. Consequently, determining an effective, economical, convenient and less harmful method than traditional chemotherapy is necessary.

Oral metronomic chemotherapy (OMCT) is the administration of continuous low-dose chemotherapeutic agents that inhibit tumour angiogenesis, promote immune-modulating effects and have tumour stem cell inhibition with fewer adverse effects [5]. Oral administration is preferred because it is convenient and economical. Studies have extensively investigated agents like cyclophosphamide, capecitabine, etoposide, pazopanib, methotrexate, celecoxib and vinorelbine. Comprehensive and impressive studies using OMCT in recurrent/metastatic breast and head-neck cancer showed good tumour control with a better safety profile [6,7].

We assess the effectiveness and safety of the cyclophosphamide and capecitabine OMCT regimen in recurrent, metastatic ovarian and cervical cancer patients who cannot financially afford anti-angiogenic agents or immunotherapy. This article was previously presented as a conference poster at the European Society of Medical Oncology (ESMO) Asia Congress 2023 held in Singapore on 2 December 2023.

## Materials and methods

This retrospective observational study from November 2021 to June 2023 was conducted jointly in the radiation and gynaecology departments at Chittaranjan National Cancer Institute, Kolkata, India. The Institutional Ethics Committee of the said institution approved the study protocol with a waiver for patient consent (approval number: CNCI-IEC-RG-2023-79). All patients more than 18 years old with histologically proven advanced/metastatic epithelial ovarian and cervical cancer (either squamous or adenocarcinoma), with measurable diseases, who were previously treated with at least one line of platinum-based chemotherapy, who had previous surgery or chemoradiotherapy for primary treatment and with adequate organ function and who were able to swallow oral medicine were included in the study. Platinum-resistant and platinum-refractory cancers were also included in this study. We included platinum-pretreated cervical cancer patients who progressed within six months of platinum (either concurrent chemoradiation or platinum-based doublet chemotherapy).

Patients with inadequate medical records and those who defaulted after starting the OMCT regimen were excluded from the study. Primary endpoints were assessments of efficacy in terms of response rate and PFS, whereas OS and safety by assessing common toxicities (haematological, altered liver enzymes, fatigue and chemotherapy-induced nausea and vomiting (CINV)) were the key secondary endpoints. Data, including baseline characteristics, histology, Eastern Cooperative Oncology Group performance status (ECOG PS), previous lines of therapy, OMCT details, responses, toxicities and survival, were collected from the hospital's medical records section.

Consecutive patients fulfilling the eligibility criteria were offered either a split course of cyclophosphamide tablets (50 mg once daily for 21 days every 28 days) and capecitabine tablets (500 mg twice daily continuously) or a fixed-dose combination of capecitabine 1800 mg tablets plus cyclophosphamide 80 mg tablets for 14 days every 21 days. These regimens were followed until disease progression or unacceptable toxicities occurred. In the split-dose regimen, capecitabine tablets were continuously given in lower doses instead of 1000-1250 mg/m^2^ twice daily to reduce the incidence of hand-foot syndrome. The fixed-dose combination was available in two strengths: 400 mg capecitabine/20 mg cyclophosphamide and 700 mg capecitabine/30 mg cyclophosphamide. Two tablets of 700/30 strength and one of 400/20 strength were given daily for 14 days. Response evaluation was performed every three cycles according to the Response Evaluation Criteria in Solid Tumours (RECIST) v1.1. Contrast-enhanced computed tomography scan (CECT) of the thorax, abdomen and pelvis was done periodically for RECIST. The overall response rate was calculated from the combined complete and partial response rates. The Common Terminology Criteria for Adverse Events (CTCAE) v5.1 were used to evaluate drug toxicities.

Statistics

Descriptive statistics were used to analyse the demographic details and the clinical and treatment variables. Kaplan-Meier plots and Cox regression were used to estimate PFS and OS. Patients who received at least six weeks of OMCT were included in the survival analysis. Data were censored on 31 June 2023. Patients lost to follow-up were censored at the time of last contact or follow-up and included in PFS and OS analysis, and the outcomes of these patients were confirmed by telephonic contact. PFS was calculated from the date of initiation of OMCT to the date of disease progression or death from any cause. The period between the dates of diagnosis and death from any cause was calculated as OS. Stata Statistical Software: Release 13 (2013; StataCorp LLC, College Station, Texas, United States) was used for statistical analysis.

## Results

Out of 34 potential patients, 24 met the eligibility criteria and were recruited for this study. The median age for this cohort was 56 years (IQ: 44-75 years). Most patients (67%) had an ECOG PS of 3 at the start of this oral therapy. There were 19 (67%) patients who had ovarian cancer with serous histology and five patients with cervical cancer. Most patients (67%) were at stage IV at the initiation of treatment. Two-thirds of patients were platinum-refractory, with around 59% of patients having previously received three lines of chemotherapy. One-third of patients received a fixed-dose regimen. Table 1 presents the demographic characteristics and treatment details.

**Table 1:** Demographic and treatment details ECOG: Eastern Cooperative Oncology Group; FIGO: International Federation of Gynaecology and Obstetrics; NOS: not otherwise specified; OMCT: oral metronomic chemotherapy

Variables	Number (n=24)	%
Age: median (in years)	56 (IQ 44-75)	
Ovarian	19	80
Cervical	5	20
ECOG		
PS2	8	33
PS3	16	67
FIGO stage		
III	8	33
IV	16	67
Histology		
High-grade serous	16	67
Squamous cell	5	21
Adenocarcinoma NOS (ovarian)	3	12
Platinum-refractory		
Yes	16	67
No	8	33
Prior chemotherapy		
First line	1	4
Second line	9	37
Third line and above	14	59
OMCT details		
Tab cyclophosphamide (50 mg)+tab capecitabine (500 mg)	16	67
Fixed dose (cyclophosphamide 80 mg+capecitabine 1800 mg)	8	33
Response		
Complete response	3	12
Partial response	16	67
Stable diseases	5	21
Dose interruption		
Yes	6	25
No	18	75

**Figure 1:**
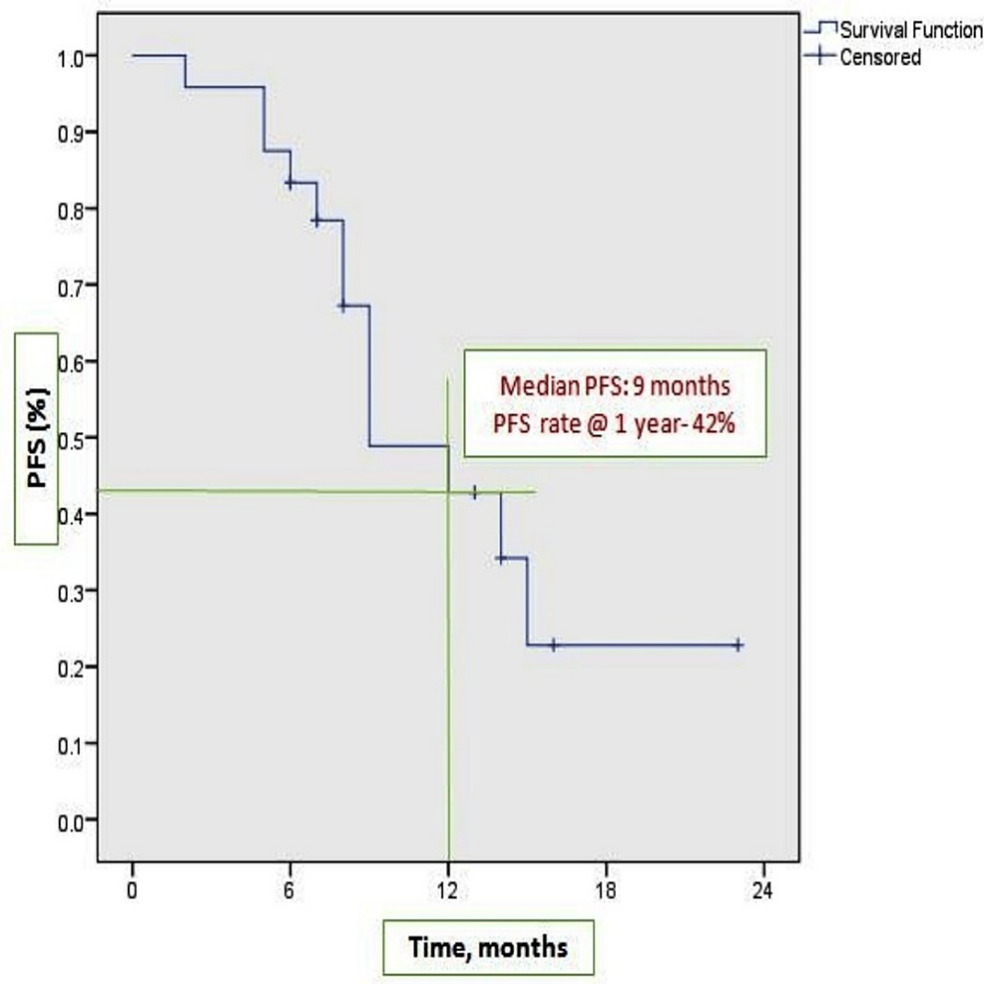
Kaplan-Meier plots of PFS PFS: progression-free survival

**Figure 2:**
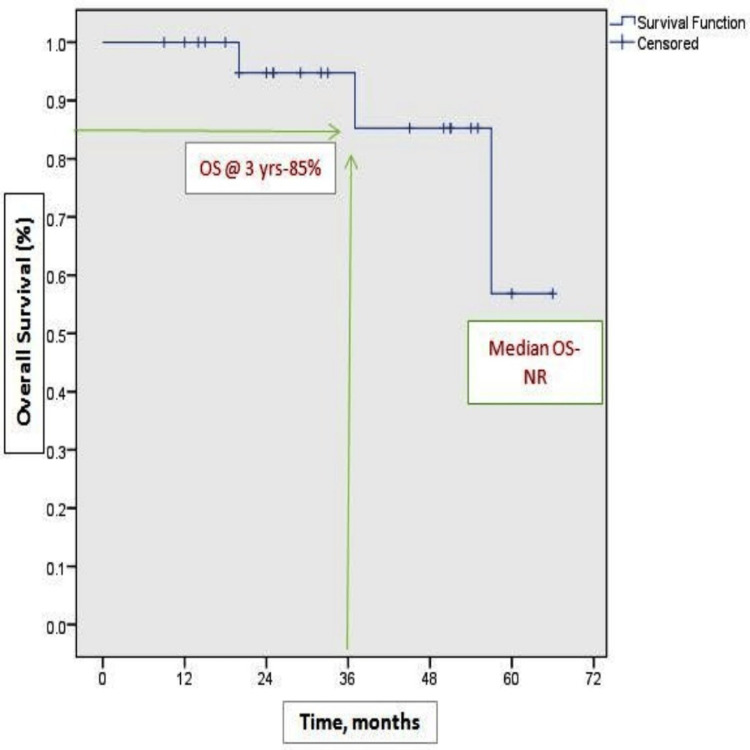
Kaplan-Meier plots of OS OS: overall survival

**Figure 3:**
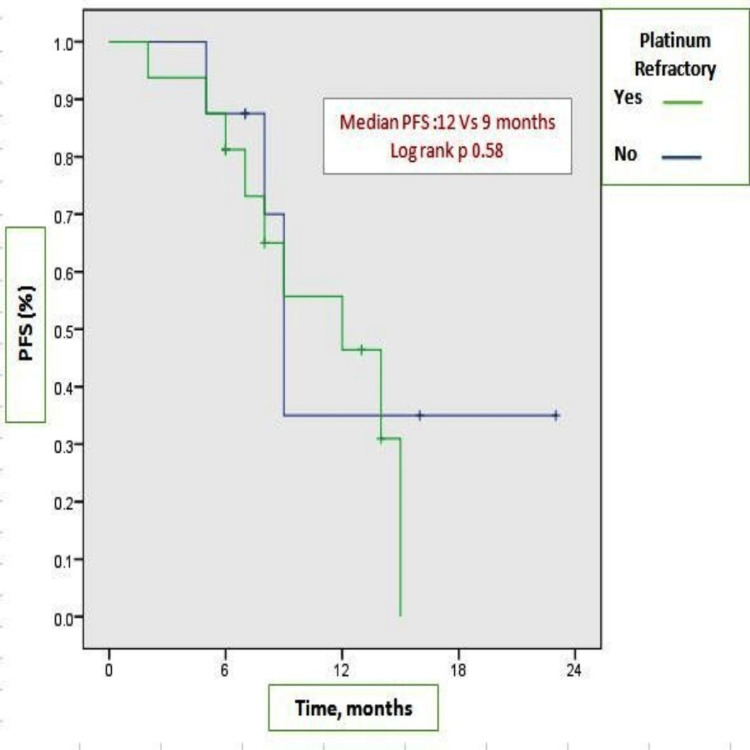
Kaplan-Meier plots of PFS among platinum-refractory group PFS: progression-free survival

At the time of analysis, 21 patients were alive. Among them, 12 patients (50%) were continuing OMCT, and six patients (25%) were on subsequent lines of chemotherapy; only three patients died. Complete response was 12%, and 67% of patients experienced partial response, with an overall response rate of 79%. Dose interruption was done in 25% of cases (Table 1). The study's median follow-up was 18 months (95% CI: 6.1-23.2). Figure 1 shows the Kaplan-Meier plots of the median PFS of nine months (95% CI: 5.2-12.7). OS was not reached due to an inadequate number of events. The three-year estimated OS rate was 85% (Figure 2). Remarkably higher median PFS was observed in the platinum-refractory group, 12 months versus 9 months (log-rank p 0.58), though not significant statistically (Figure 3).

Common adverse events were fatigue (79%), elevated liver enzymes (33%), CINV (80%), anaemia (83%) and neutropenia (29%). Adverse events are mainly grade I or II. Grade III CINV occurred in only two patients, though dose interruption occurred in 25%. Toxicities according to severity are summarised in Table 2.

**Table 2:** Toxicity details

Toxicity	Grade I	Grade II	Grade III	Total (%)
Anaemia	13	7	0	83%
Nausea and vomiting	11	6	2	80%
Fatigue	10	9	0	79%
Elevated liver enzymes	8	0	0	33%
Neutropenia	5	2	0	29%

## Discussion

This study assessed the effectiveness of the OMCT regimen containing cyclophosphamide and capecitabine in advanced, recurrent/metastatic epithelial ovarian and cervical cancer. Our results demonstrate that this regimen is associated with very good objective response and PFS in heavily pretreated patients. Objective response rate (ORR) is 79%, with a median PFS of nine months.

Advanced gynaecological cancer patients whose disease has progressed or become refractory after multiple lines of chemotherapy are of poor performance status and not fit for further IV chemotherapy. OMCT is a convenient, economical and safe option for them. The main mechanisms behind metronomic therapy are its anti-angiogenesis and effect on regulatory T cells [8,9]. Angiogenesis has been established as a hallmark of tumour development, growth and metastasis. Many studies of oral cyclophosphamide have been evaluated in recurrent ovarian cancer alone or in combination with etoposide, hormonal agents or anti-angiogenic agents. However, those were retrospective analyses or phase I/II trials; the results may have been influenced by small sample sizes and different inclusion or exclusion criteria [10]. A meta-analysis by Huang et al. assessing the effectiveness of oral cyclophosphamide in recurrent or refractory epithelial ovarian cancer (EOC) showed that the pooled ORR and disease control rate (DCR) were 25% and 61%, respectively [11]. The PFS and OS were 4.29 months and 11.26 months, respectively [11]. This also showed that in subgroup analysis, when cyclophosphamide was combined with bevacizumab/pazopanib, the ORR, DCR, PFS and OS were better, with the evaluated outcomes as 42% and 82% and 7.32 months and 17.35 months, respectively [11]. A few retrospective studies combining low-dose cyclophosphamide with bevacizumab showed a moderate response rate associated with bevacizumab-related toxicities like hypertension, diarrhoea, haematuria, thrombosis, fistula or perforation [12,13]. Gulia et al. showed that combining cyclophosphamide and pazopanib in platinum-refractory ovarian cancer resulted in a median PFS of 5.5 months and a median OS of 9.5 months [14]. Common adverse events (grade III or IV) were fatigue (25%), diarrhoea (15%), hand-foot syndrome (10%), mucositis (10%), transaminitis (5%) and hypertension (5%) [14]. Similarly, per Vasey et al., capecitabine, when used as monotherapy, had a response rate of 29%, but 14% of patients experienced grade III hand-foot syndrome and 10% of patients suffered from grade III vomiting due to a higher capecitabine dose [15]. Chakrabarti et al. [16] showed bevacizumab combined with capecitabine results in a higher PFS of 10.51 months and OS of 20.53 months, though, in this study, a higher percentage of patients had good performance status and around 50% of patients had received only one line of chemotherapy. Four patients experienced grade III hypertension. Grade II proteinuria was seen in five patients, and one patient experienced bowel perforation [16].

Comparably, Tomao et al. suggested a promising role for capecitabine both as monotherapy in patients with platinum-resistant cervical cancer and in combination with cisplatin in chemotherapy-naïve patients with metastatic or recurrent cervical cancer [17]. Garcia et al. phase II study evaluated the role of capecitabine as a single agent in a population of 26 chemo-naïve patients with advanced or recurrent cervical carcinoma [18]. The study was stopped prematurely because of futility; the drug was claimed to be ineffective, even though a 15.4% ORR and a 34.6% stable disease rate (SDR) were documented [18]. Recently published, Maltese et al.'s research showed a clinical benefit rate of 57% and an ORR of 34.2% using capecitabine in recurrent cervical cancer [19]. A pilot phase II study on a population of 30 stage IVB or recurrent cervical cancer patients who had received >1 chemotherapy regimens reported a 43.3% ORR with a median time to progression of 4.4 months and a median OS of 10.2 months with S-1 (an oral anticancer drug comprised of tegafur, gimeracil and oteracil) at a dose of 40-60 mg/m^2^ twice daily for six weeks [20]. The most frequent grade III or IV adverse events were neutropenia (13.3%) and nausea (16.7%).

A survey of the available literature encouraged us to use combination instead of monotherapy OMCT in lower dose levels and avoid anti-angiogenic agents like bevacizumab or pazopanib, as our population was mainly of poor performance status and heavily pretreated. The use of anti-angiogenic agents is financially challenging, producing modest responses with higher grade III or IV hypertension, diarrhoea, haematuria, fistula, thrombosis and perforation. These agents seem to be poorly tolerated in recurrent disease.

Both cyclophosphamide and capecitabine are effective in ovarian and cervical cancer, an inexpensive combination without synergistic toxicities. There is less need for hospital visits and supportive investigations with this regimen, which is helpful in high-volume resource-limited settings. This combination was well tolerated, with mainly grade I or II toxicities. The most common adverse events were fatigue, anaemia, CINV and elevated liver enzymes. Dose reduction of capecitabine helped alleviate hand-foot syndrome and diarrhoea problems. Interruption of OMCT was required in 25% of cases due to toxicities, but no patient stopped treatment as a result. Toxicities were managed by supportive care, and OMCT usually restarted within two to three weeks.

In our study, the median PFS in platinum-refractory patients was 12 months, which is much higher than the Avastin Use in Platinum-Resistant Epithelial Ovarian Cancer (AURELIA) trial [21]; it reported a median PFS of 6.7 months with the combination of bevacizumab and chemotherapy among platinum-refractory patients who had received two or fewer prior lines of chemotherapy. Our analysis showed that ORR was 79% with 67% partial response, which was sustainable for over six months and demonstrably higher than capecitabine monotherapy in recurrent cervical cancer per the literature review. In our study, with a median follow-up of 18 months, only three patients died, and 12 patients were continuing OMCT.

Major limitations of this study are that it is retrospective, has a small sample size, has short follow-up and has inadequate events leading to higher OS. The inclusion of both cervical and ovarian cancer patients may have altered the outcome. Despite those limitations, this OMCT regimen shows better ORR and PFS in comparison with other OMCT regimens with or without anti-angiogenic agents used in advanced and recurrent gynaecological cancer.

## Conclusions

The anti-angiogenic effect of oral metronomic drugs proved its efficacy in several solid tumours. However, large-scale prospective data are still needed. The OMCT regimen used in this study was well tolerated, with remarkable clinical benefits and survival outcomes in advanced recurrent/metastatic gynaecological cancer. Using cyclophosphamide and capecitabine in reduced doses improves the safety profile without compromising disease control. Outpatient clinic-based treatment and reduced hospital admission with minimal interventions for supportive care improve patient compliance. Accordingly, OCMT can be a very convenient and economical option for financially challenged, heavily pretreated, poor-performance-status patients in resource-limited settings.
